# Zika Virus Growth in Human Kidney Cells Is Restricted by an Elevated Glucose Level

**DOI:** 10.3390/ijms22052495

**Published:** 2021-03-02

**Authors:** Alawiya Reslan, Juliano G. Haddad, Liadrine Moukendza Koundi, Philippe Desprès, Jean-Loup Bascands, Gilles Gadea

**Affiliations:** 1 Unité Mixte Processus Infectieux en Milieu Insulaire Tropical, Plateforme Technologique CYROI, Université de la Réunion, INSERM U1187, CNRS UMR 9192, IRD UMR 249, 94791 Sainte Clotilde, La Réunion, France; alawiyareslan@hotmail.com (A.R.); juliano.haddad@univ-reunion.fr (J.G.H.); liadrinekoundi@yahoo.fr (L.M.K.); philippe.despres@univ-reunion.fr (P.D.); 2 Unité Mixte Diabète athérothrombose Thérapies Réunion Océan Indien, Plateforme Technologique CYROI, Université de la Réunion, INSERM U1188, 94791 Sainte Clotilde, La Réunion, France

**Keywords:** flavivirus, Zika virus, diabetes, high glucose, kidney cells, viral infection, viral replication

## Abstract

Mosquito-borne Zika virus (ZIKV) became a real threat to human health due to the lack of vaccine and effective antiviral treatment. The virus has recently been responsible for a global outbreak leading to millions of infected cases. ZIKV complications were highlighted in adults with Guillain–Barré syndrome and in newborns with increasing numbers of congenital disorders ranging from mild developmental delays to fatal conditions. The ability of ZIKV to establish a long-term infection in diverse organs including the kidneys has been recently documented but the consequences of such a viral infection are still debated. Our study aimed to determine whether the efficiency of ZIKV growth in kidney cells relates to glucose concentration. Human kidney HK-2 cells were infected with different ZIKV strains in presence of normal and high glucose concentrations. Virological assays showed a decrease in viral replication without modifying entry steps (viral binding, internalization, fusion) under high glucose conditions. This decrease replication was associated with a lower virus progeny and increased cell viability when compared to ZIKV-infected HK-2 cells in normal glucose concentration. In conclusion, we showed for the first time that an elevated glucose level influences ZIKV replication level with an effect on kidney cell survival.

## 1. Introduction

Mosquito-borne Zika virus (ZIKV) belonging to the *flavivirus* genus (*Flaviviridae* family), was first isolated in 1947 from a sentinel monkey in the Zika forest (Uganda). ZIKV circulated in Africa and Asia for 70 years, only causing sporadic cases. In 2007, the first ZIKV outbreak was reported on Yap Island, affecting 70% of inhabitants [1]. From this date, ZIKV spread to the Pacific Islands, and South and Central America, and then became a major health concern worldwide [2]. Currently, to the best of our knowledge, there is no vaccine for preventing ZIKV infection. A vast majority of infections remain asymptomatic or cause mild symptoms. However, ZIKV infections can also account for severe diseases including Guillain–Barré syndrome in adults and congenital neurological disorders [3,4,5]. Rapid diffusion around the world and drastic epidemiological changes are witnesses of ZIKV adaptation to humans. Indeed, supporting evidence depicts ZIKV’s ability to disseminate from human to human, through non-vectorial routes [6,7,8]. These new behaviors highlight ZIKV enlarged cellular tropism as well as a long-term viral infection in target tissues.

Like other flaviviruses, ZIKV contains a genomic single-stranded positive ribonucleic acid (RNA) encoding a single large polyprotein that is subsequently cleaved by cellular and viral proteases into three structural proteins (C, prM/M, and E) and seven nonstructural proteins (NS1 to NS5). The structural proteins form the viral particle surrounding genomic viral RNA. They are responsible for viral entry into host cells. Viral particle first binds to cellular attachment factors and receptors, leading to virion internalization primarily through a clathrin-mediated endocytic pathway [9] and fusion of viral and cellular membranes occurs in endosomes [10]. The nonstructural proteins are responsible for virus replication, assembly, and escape from the host immune system [11]. 

Kidney has been recognized as a target for various flaviviruses including ZIKV [12]. Detection of viral RNA in urine up to two weeks after acute ZIKV infection has been reported [13]. Such findings sustain the hypothesis that the kidney could contribute to the pathophysiology of ZIKV infection as a potential viral reservoir. To date, the impact of ZIKV infection on kidney biology has not been clearly understood. It is of note that long-term consequences of urinary tract shedding of ZIKV are still unknown especially in the context of pre-existing chronic pathology such as elevated glucose levels in humans. Regardless of the country’s income level, the prevalence of diabetes mellitus has dramatically increased worldwide [14]. At least a quarter of diabetic patients develop diabetic kidney disease (DKD) which represents the main cause of chronic kidney disease leading to end-stage kidney disease in both type 1 and type 2 diabetes [15,16]. The severe kidney damage in diabetic patients remains the main determinant of the dramatically increased risk of death [17]. It has been recently demonstrated a strong permissiveness of cultured renal cells to ZIKV infection [18]. Notwithstanding, the impact of glucose concentration on ZIKV infection of the kidney and its potential consequences have not been explored. In the present study, the ability of ZIKV to replicate in human kidney cells was assessed in the condition of elevated glucose level. We demonstrated that exogenous glucose has an impact on the efficacy of ZIKV growth in vitro.

## 2. Results and Discussion

### 2.1. High-Glucose Condition Restricts ZIKV Growth in HK-2 Cells

**Figure 1:**
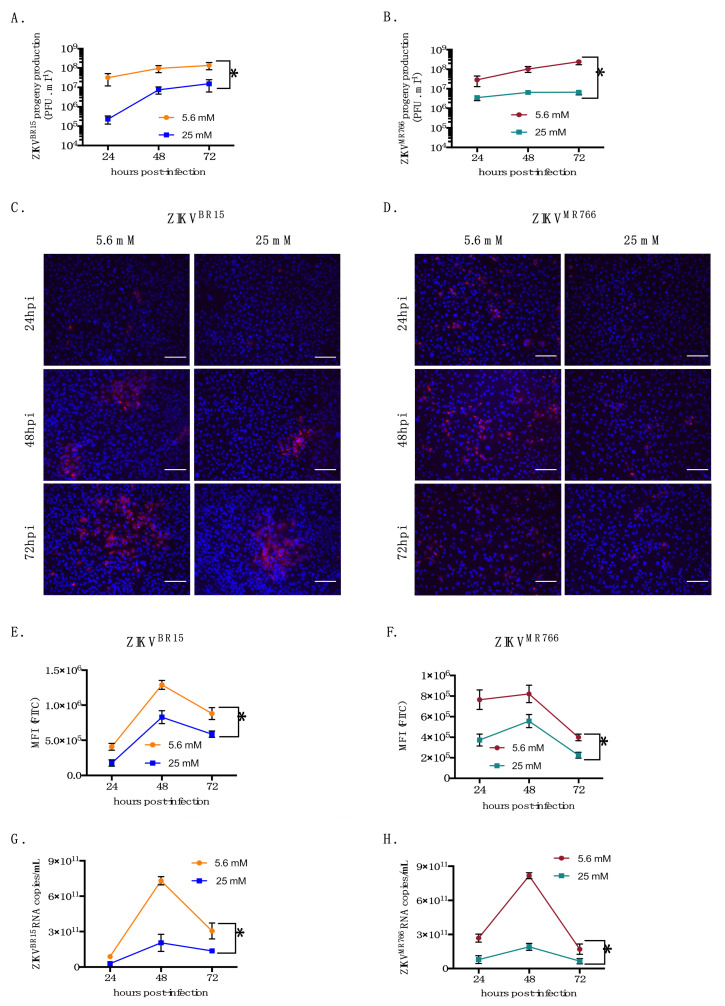
Exogenous glucose interferes on ZIKV growth in HK-2 cells. HK-2 cells were infected with ZIKV^BR15^ and ZIKV^MR766^ at MOI of 1. ZIKV progeny production in cell culture supernatants of HK-2 infected cells with ZIKV^BR15^ (**A**) and ZIKV^MR766^ (**B**) were quantified by plaque-forming assay at 24, 48, and 72 hpi. Detection of intracellular E protein in ZIKV^BR15^ (**C**) and ZIKV^MR766^ (**D**) infected HK-2 cells at 24, 48, and 72 hpi by immunofluorescence using anti-E pan flavivirus monoclonal antibody (mAb) 4G2-Alexa Fluor 596. The ZIKV E protein (red) and nuclei (blue) were visualized by fluorescence microscopy. In (**E**,**F**) flow cytometry was performed and E protein was detected in ZIKV^BR15^ and ZIKV^MR766^ HK-2 infected cells, respectively using anti-E pan flavivirus mAb 4G2. The number of viral genomic RNA in ZIKV^BR15^ (**G**) and ZIKV^MR766^ (**H**) -infected HK-2 cells was determined at 24, 48, and 72 hpi by RT-qPCR. Results are shown as means ± SEM of three independent experiments. Two-way ANOVA test shows that differences are statistically significant (* *p* < 0.05). Scale bars represent 100 µm.

Human immortalized kidney HK-2 cells were routinely cultured in 5.6 mM glucose medium. In prior experiments, HK-2 cells were high-glucose adapted over a 10-day period to allow metabolic reprogramming. Cells were first grown in 15 mM glucose medium for 3 days then shifted in 25 mM glucose medium for another 7 days. As HK-2 cell proliferation could effectively be sensitive to experimental conditions [19], we performed growth kinetics using 5.6 mM (normal growth condition) and 25 mM (adapted growth condition) glucose, hereafter designed as low- and high-glucose conditions, respectively. Our results show that, over a 72-h period, HK-2 cells grow slightly but significantly faster in high-glucose conditions (Supplementary Figure S1). Accordingly, cell plating along the study was adjusted to avoid multiplicity of infection (MOI) bias during infections. First, to test whether glucose concentration could impact ZIKV infection, we analyzed viral progeny over 72 h. HK-2 cells were infected at an MOI of 1 with two different molecular clones of ZIKV whose sequences derived from the epidemic Brazilian BeH819015 strain (hereafter called ZIKV^BR15^) and the laboratory-adapted MR766^NIID^ strain (hereafter called ZIKV^MR766^) [20,21]. The level of 5.6 mM glucose in the growth medium was used as a control. Our data show that high glucose delayed viral progeny at 24 h post-infection (hpi) and still reduced it by 1 log_10_ at 48 and 72 hpi for ZIKV^BR15^ (Figure 1A) and up to 2 log_10_ at 72 hpi for ZIKV^MR766^. To eliminate a potential role for osmolarity in the observed phenomenon, HK-2 cells were adapted with mannitol as it was done for glucose. Supplementary Figure S2A,B show no difference in progeny production with both viral clones suggesting that osmolarity is not the driving force of the observed restriction, but rather the glucose concentration. Then, we stained infected cells with 4G2 monoclonal antibody, which recognizes a highly conserved conformational epitope within the fusion loop sequence of most flaviviruses, including ZIKV. This staining allowed us to determine cell capacity to produce mature virions. HK-2 cells were infected, stained, and analyzed by immunofluorescence and flow cytometry. Representative images captured for the three timepoints indicate that staining is observed in a significantly higher number of cells for the normal condition of glucose (Figure 1C,D). These results were quantitatively confirmed by flow cytometry showing that the mean fluorescence intensity (MFI) of infected cells was decreased in high-glucose conditions (Figure 1E,F). These stainings demonstrate that high glucose impairs the production of mature infectious particles. In addition, to determine whether observed differences in the production of mature infectious particles could be correlated with differences in viral RNA production within HK-2 cells, quantitative reverse transcription-polymerase chain reaction (RT-qPCR) were performed on total cellular RNA. Viral RNA were analyzed and quantified using a standard curve as previously described [22]. Viral copy numbers were plotted over a 72-h period. Results in Figure 1G,H unambiguously show that ZIKV^BR15^ and ZIKV^MR766^ infection of HK-2 cells cultured under high-glucose conditions produced less viral genome copies.

**Figure 2:**
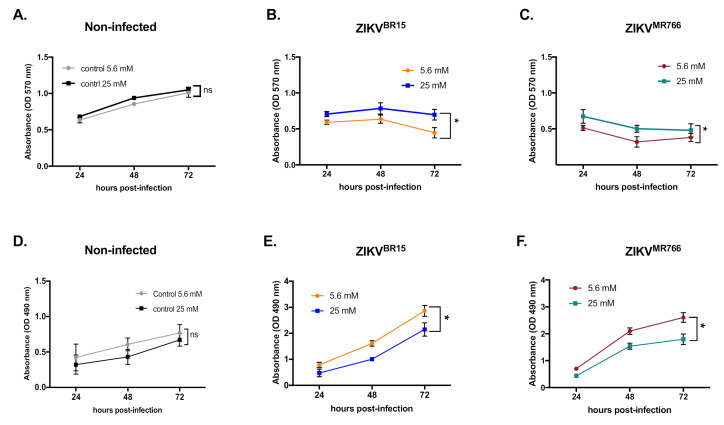
ZIKV-mediated cytopathic effects under high-glucose condition. The metabolic activity of non-infected HK-2 cells (**A**) or infected with ZIKV^BR15^ (**B**) and ZIKV^MR766^ (**C**) at MOI of 1 in low- and high-glucose conditions was determined at different time-points of infection using 3-[4,5-dimethylthiazol-2-yl]-2,5-diphenyltetrazolium bromide (MTT) assay. Analysis of Lactate Dehydrogenase (LDH) release was performed to evaluate the membrane integrity of uninfected (**D**), ZIKV^BR15^ (**E**), and ZIKV^MR766^ (**F**) -infected HK-2 cells. Data were obtained by measuring the absorbance at 570 nm and 490 nm for MTT and LDH assays respectively, with a background subtraction of the absorbance at 690 nm. Results are shown as means ± SEM of three independent experiments. Two-way ANOVA test demonstrates that differences are nonsignificant (ns) or statistically significant (* *p* < 0.05).

Then we assayed whether the viral restriction observed in high glucose conditions could be associated with host-cell physiology. We first tested the metabolic activity using 3-[4,5-dimethylthiazol-2-yl]-2,5-diphenyltetrazolium bromide (MTT) assay as an indicator of cell health. We observed no significant differences in MTT reduction in control cells (Figure 2A). In contrast, the metabolic activity of ZIKV-infected cells was higher in high-glucose than in low-glucose conditions (Figure 2B,C), which probably reflects a less active ZIKV replication. We next investigated whether a loss of cell integrity in high-glucose conditions could account for ZIKV restriction in HK-2 cells. To test this hypothesis, we quantified in culture supernatants the release of lactate dehydrogenase (LDH), which is a key feature of cells undergoing apoptosis, necrosis, or other forms of damage. LDH activity in culture supernatants of uninfected HK-2 cells indicates that, in our culture conditions, glucose concentration per se did not affect cell integrity (Figure 2D). In contrast, in ZIKV-infected cells, LDH activity was significantly lower in high-glucose conditions, indicating apparent protection of HK-2 cells against ZIKV^BR15^ and ZIKV^MR766^ infection (Figure 2E,F). All these data suggest that, in our culture conditions, high glucose restricts ZIKV infection of HK-2 cells and that ZIKV restriction by high glucose seems to be a general feature of ZIKV and not specific to epidemic strains.

### 2.2. High-Glucose Condition Does Not Affect Early Steps of ZIKV Infection in HK-2 Cells

**Figure 3:**
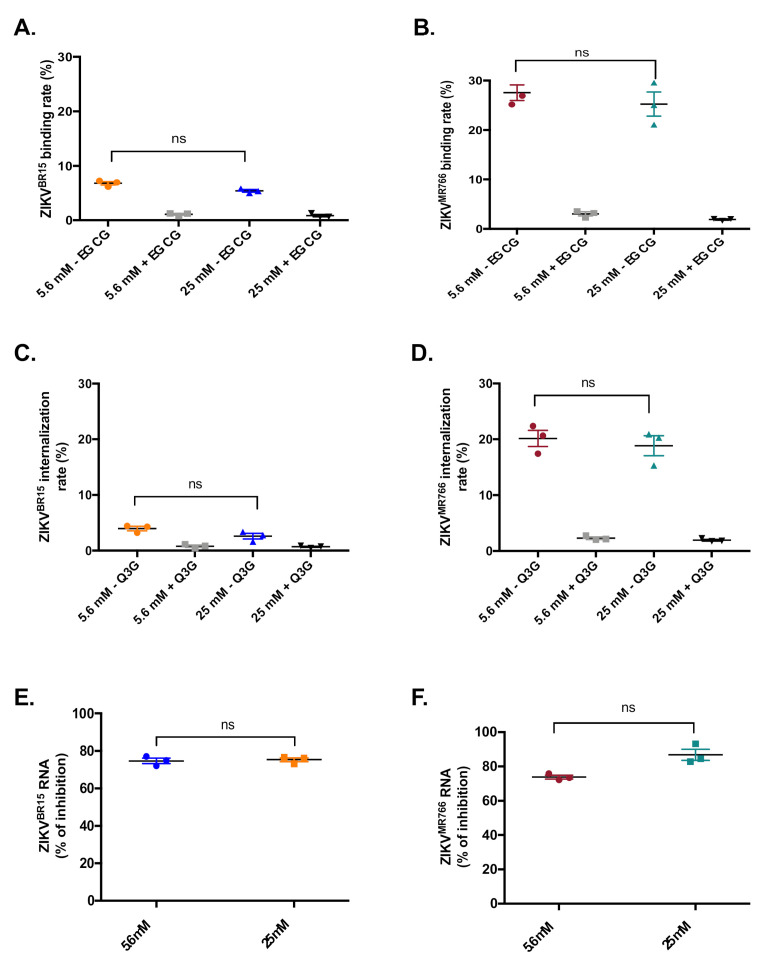
High glucose level does not affect ZIKV entry in HK-2 cells. For virus binding assay (**A**,**B**), HK-2 cells were infected for 1 h at 4 °C with ZIKV^BR15^ and ZIKV^MR766^, respectively, at MOI of 1. The epigallocatechin gallate (EGCG) described to inhibit the ZIKV attachment step was used as a positive control at 100 µM. The amount of viral particles attached to the cell surface was quantified by RT-qPCR. For the virus internalization assay, HK-2 cells were incubated 1 h with ZIKV^BR15^ (**C**) and ZIKV^MR766^ (**D**) at 4 °C and then shifted to 37 °C for 1 h. Isoquercitrin (Q3G) known to inhibit the ZIKV internalization step was used as a positive control at 200 µM. The amount of viral particles internalized into HK-2 cells was determined by RT-qPCR. For virus fusion assay, HK-2 cells were incubated with ZIKV^BR15^ (**E**) and ZIKV^MR766^ (**F**) at MOI of 1 at 4 °C then shifted to 37 °C for 1 h. Chloroquine was added for 2 h at 37 °C. Viral genomic RNA was measured by RT-qPCR. Results are expressed as the percentage of viral fusion inhibition by chloroquine under both glucose conditions. Results are shown as means ± SEM of three independent experiments. Mann-Whitney test was used for statistical analysis (n.s. = not significant).

The observed impairment of viral growth in high-glucose conditions could occur at different stages of the ZIKV infectious cycle. To determine which stage could be affected, we first focused our study on virus entry. ZIKV entry is a multistep process that requires the viral particle to bind onto the cell surface receptor, then to get internalized through clathrin-dependent endocytosis, and to finally fuse its membrane with endosomal membranes [23]. Each of these steps could be specifically investigated. As such, we first tested viral binding. Cells were infected with either ZIKV^BR15^ or ZIKV^MR766^ for 1 h at 4 °C to avoid viruses to get endocytosed. Unbound viral particles were washed off, while bound viruses were quantified by RT-qPCR. Figure 3A,B show that, in both glucose conditions, the numbers of ZIKV^BR15^ or ZIKV^MR766^ particles bound onto HK-2 cells (expressed as viral RNA copies) were not statistically different. To rule out any experimental artifacts, we used, as a positive control, epigallocatechin gallate (EGCG), a natural compound that inhibits ZIKV binding onto host cells [24]. Indeed, EGCG significantly inhibited viral binding whatever the glucose concentration in the culture medium (Figure 3A). These experiments indicate that an elevated glucose concentration doesn’t affect ZIKV binding onto HK-2 cells. 

Next, we investigated the internalization of viral particles into the host cells. Cells were infected with ZIKV^BR15^ or ZIKV^MR766^ for 1 h at 4 °C. Then unbound viral particles were removed and internalization was allowed to occur for 1 h at 37 °C. Uninternalized particles were washed off using citrate buffer and internalized viruses were quantified by RT-qPCR. Again, the numbers of internalized particles were not statistically different whatever the glucose condition (Figure 3C,D). As a positive control, we used isoquercitrin (Q3G), a natural flavonoid that we previously published to prevent ZIKV internalization [25]. As expected, Q3G exhibited a strong inhibition of ZIKV^BR15^ internalization in both glucose conditions (Figure 3C,D). These results indicate that glucose does not either affect ZIKV internalization into HK-2 cells. 

Finally, we looked to test whether high glucose level could affect the fusion step. The fusion of the viral and cellular membranes occurs in endosomes after conformational changes of ZIKV envelope proteins triggered by low pH [10]. We previously published that chloroquine, a weak base that inhibits endosome acidification, can restrict ZIKV infection by blocking membrane fusion [22]. HK-2 cells were infected for 1 h at 4 °C and internalization was allowed to occur for another 1 h at 37 °C. Then infected cells were treated or not with 100 µM chloroquine. Because long-term treatments are cytotoxic, chloroquine was removed after 2 h (time after which viruses must have fused with endosomal membranes or are gone into the lysosomal compartment for degradation). Viral RNA contents in ZIKV-infected cells were quantified 30 hpi by RT-qPCR. Results were expressed as the percentage of fusion inhibition by chloroquine (Figure 3E,F). The glucose concentration of the culture medium does not affect viral fusion. 

Flaviviruses are well known to hijack host-cell translation machinery for the production of new virus progeny [26]. Indeed, if defaults are observed in the cellular translation machinery, this might affect viral infection. Glucose control of translation has been well documented, both acting on efficiency and capacity of translation [27]. To test the glucose impact on translation in our system model, we use a GFP reporter system. HK-2 cells were transfected with increasing amounts of GFP-expressing plasmid. Then, GFP expression was analyzed by flow cytometry 24 h post-transfection. 5.6 mM glucose was used as a control condition. Dose-response curves were not statistically different between both glucose conditions (Supplementary Figure S3). This result suggests that, in our experimental conditions, translation is not significantly affected by glucose levels and, consequently, that restriction of ZIKV infection in high-glucose conditions is probably not linked to translation defaults. Altogether, these results indicate that the restriction of ZIKV infection observed with high glucose does not seem to occur at viral entry nor the translation level.

### 2.3. High Glucose Level Impacts ZIKV Replication in HK-2 Cells

**Figure 4:**
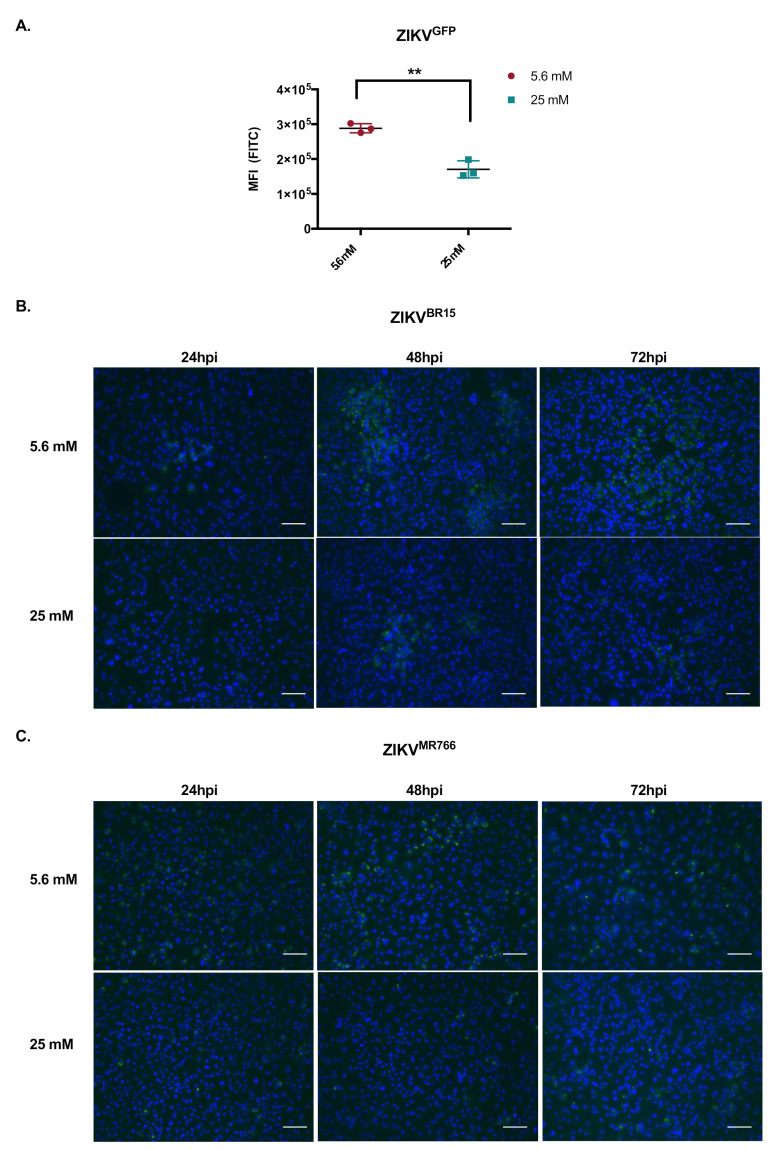
Effect of high glucose on ZIKV replication step. HK-2 cells were infected with ZIKV^GFP^, ZIKV^BR15,^ and ZIKV^MR766^ as indicated at an MOI of 1 in low- and high-glucose conditions. (**A**) GFP-expression of ZIKV^GFP^ -infected HK-2 cells were analyzed by flow cytometry at 24 hpi. In (**B**,**C**) double-stranded RNA (dsRNA) of ZIKV^BR15^ and ZIKV^MR766^ was detected by immunofluorescence at 24, 48, and 72 hpi using J2 mAb. ZIKV dsRNA (green) and nuclei (blue) were visualized by fluorescence microscopy. Results are shown as means ± SEM of three independent experiments. Mann-Whitney test shows that the difference between these two conditions are statistically significant (** *p* < 0.01). Scale bars are 100 µm.

All the above-described data suggest that ZIKV restriction by high glucose concentration could take place in HK-2 cells at the viral replication level. To test this hypothesis, we took advantage of the MR766 based GFP-reporter ZIKV (ZIKV^GFP^), whose GFP expression, as part of the viral polyprotein, is a direct reporter of viral propensity to replicate [20]. HK-2 cells were infected at an MOI of 1 and green fluorescent protein (GFP) expression was analyzed by cytometry at 24 hpi, as GFP accumulation was cytotoxic at later time points (data not shown). The MFI of ZIKV^GFP^-infected HK-2 cells was significantly lower in the high-glucose condition (Figure 4A). To confirm these results, infected HK-2 cells were immunostained with J2 monoclonal antibody, which only recognizes double-stranded RNA, a key feature of RNA virus replication. Figure 4B (ZIKV^BR15^) and C (ZIKV^MR766^) show that immunosignal was lower in high glucose concentration. These results support that ZIKV infection of HK-2 cells is impaired by high glucose at the viral replication level.

### 2.4. Concluding Remarks and Discussion

From a general point of view, flavivirus replication is based on host cell machinery hijacking. However, although several clinical studies have identified diabetes as a risk factor, no experimental study has ever reported if a hyperglycemic environment could influence flavivirus replication. In the present study, we aimed to investigate the impact of high glucose (25 mM) on ZIKV infection of HK-2 kidney cells. Our results show that high glucose can restrict ZIKV infection of HK-2 cells, which was illustrated by a decrease in progeny production, mature envelope protein expression, and intracellular viral RNA. Analysis of the ZIKV life cycle demonstrated that restriction occurs during the replication without affecting early infection stages (i.e., binding to cellular receptors, internalization or fusion of viral and cellular membranes, or even protein translation). Furthermore, our data emphasize that ZIKV restriction by high glucose is not a hallmark of epidemic strains that were responsible for the recent outbreak, but is a general feature of ZIKV. We proposed three hypotheses to explain, at least in part, ZIKV restriction by high glucose. 

Might glucose-mediated protein kinase C (PKC) activation play a role in ZIKV restriction in renal cells? The role of PKC activation on flavivirus infection is still a matter of debate. PKC phosphorylation of the C viral protein has been described as the key regulator of apoptosis induction during West Nile virus (WNV) infection [28], and inhibition of all PKC isoforms affected WNV entry into mosquito cells or replication in green monkey cells [29,30]. Conversely, Dengue virus replication was enhanced after in vitro inhibition with a specific chemical inhibitor of classical and novel PKCs [31]. Although PKC is either implicated in viral inhibition or promotion, the latter studies have been developed in normal glucose conditions. Furthermore, glucose has been reported to increase total diacylglycerol (DAG) content in many cell types including kidney cells [32]. This increase in total DAG content leads in part to the activation of PKC family members [33,34]. Although the role of PKC family members have not been documented in the context of ZIKV infection, we believe that further investigations on PKC activation in HK-2 cells under high glucose condition might help to better understand the ZIKV restriction mechanism. 

Is glucose-induced endoplasmic reticulum stress involved in ZIKV restriction? Excessive concentrations of glucose can also modulate the activity of the endoplasmic reticulum (ER) which is the central organelle for the folding and post-translational modifications of soluble proteins. During stress conditions, proteins that fold improperly accumulate in the ER leading to an ER stress response, which allows the ER folding machinery to catch up with the backlog of misfolded proteins. It has been reported that hyperglycemia can induce ER stress in renal epithelial cells, and that chronic hyperglycemia induces an adaptive, protective ER stress response [35]. ZIKV replication takes place in ER invaginations of infected cells and leads to an accumulation of viral proteins [36]. However, it has recently been shown that ZIKV infection of lung epithelial cells leads to unresolved and persistent ER stress to the benefit of viral growth [37]. Whether ZIKV is unable to hijack the ER stress response induced by high glucose to facilitate its own replication is a key question that is required to be addressed. 

Is ZIKV restriction linked to hyperglycemia-related nuclear factor erythroid 2–related factor 2 (Nrf2) broad antioxidant? Nrf2 transcription factor is a master regulator of endogenous antioxidant status. As oxidative stress plays an important role in the pathogenesis and progression of diabetes, activation of Nrf2 by agonist has been suggested as a potentially effective method for diabetes treatment [38]. More specifically, hyperglycemia-induced oxidative stress and accelerated renal injury were more serious in streptozotocin-treated Nrf2 knockout mice than those in wild-type controls, indicating a beneficial effect of Nrf2 on diabetic nephropathy [39,40,41]. Given that Nrf2 agonist has recently been identified as a broad inhibitor of viral replication, including ZIKV [42], it would be interesting to investigate whether ZIKV restriction in high glucose conditions relates to Nrf2 functions.

There is mounting evidence that flaviviruses have ability to establish persistence in human organs as it has been observed with WNV and now ZIKV [11,43]. Studies by Murray and colleagues postulated kidneys as a preferred site for the establishment of persistent flavivirus infections following the detection of WNV RNA in urine [11]. Such persistence has been associated with kidney injuries [12]. In agreement with these findings, our results would suggest that high glucose, by protecting cells from massive infection and cytotoxicity, could favor ZIKV persistence in the kidney and putative consecutive injuries. Such interpretation warrants a long-term follow-up of ZIKV patients with a history of chronic hyperglycemia.

## 3. Materials and Methods 

### 3.1. Cells, Virus, and Reagents

Human kidney proximal tubular HK-2 cells (CRL-2190, ATCC, Manassas, VA, USA) were cultured in Dulbecco’s modified Eagle’s medium (DMEM) supplemented with low D-glucose (5.6 mM), 10% fetal bovine serum (FBS), 2 mmol L^−1^ L-Glutamine, 100 U/mL penicillin, and 0.1 mg/mL streptomycin and 0.5 µg mL^−1^ of fungizone (Amphotericin B) (PAN Biotech, Aidenbach, Germany) at 37 °C under 5 % CO_2_. In prior experiments, cells were adapted to high glucose (25 mM) to allow metabolic reprogramming. Adaptation consisted to expose HK-2 cells to 15 mM D-glucose for 3 days and to 25 mM glucose for another 7 days. Vero cells (CCL-81, ATCC, Manassas, VA, USA), were maintained at 37 °C under 5% CO_2_ in Minimal essential medium (MEM), supplemented with 5% heat-inactivated Fetal Bovine Serum (FBS), 1 mmol L^−1^ sodium pyruvate, 2 mmol L^−1^ L-Glutamine, 0.5 µg mL^−1^ of fungizone, 0.1 mg mL^−1^ of streptomycin, and 100 U mL^−1^ of penicillin. Mouse anti-E monoclonal antibodies 4G2 and 4G2-Alexa Fluor 596 were purchased from RD Biotech (Besancon, France) and mouse anti-double-stranded RNA monoclonal antibodies J2 from (SCICONS) Donkey anti-mouse Alexa Fluor 488 secondary antibody from Invitrogen (Carlsbag, CA, USA). Epigallocatechin gallate (EGCG), Isoquercitrin (Q3G), and Chloroquine phosphate were purchased from Sigma-Aldrich (Saint-Louis, MO, USA). ZIKA virus (ZIKV) molecular clones (ZIKV^BR15^, GenBank accession number KU365778 and ZIKV^MR766^, GenBank accession number LC002520) were obtained using the Infectious Subgenomic Amplicon method as previously described [21,25]. Recombinant ZIKV expressing the GFP reporter gene (ZIKV^GFP^) was previously described [20].

### 3.2. Plaque-Forming Assay

Viral progeny production was quantified by standard plaque-forming assay. Vero cells were seeded in 24-well plates. The next day, cells were inoculated with 0.2 mL of ten-fold dilutions of virus samples. After 2 h of incubation at 37 °C, 0.3 mL of MEM culture medium supplemented with 5% FBS and 1% carboxymethylcellulose (Sigma-Aldrich, Saint-Louis, MO, USA) was added to cells. The medium was removed at 96 hpi and cells were washed twice with phosphate-buffered saline (PBS), fixed with paraformaldehyde (PFA) 3.7%, and stained with 1% crystal violet (Sigma-Aldrich, Saint-Louis, MO, USA) diluted in 20% ethanol. Viral titers were presented as plaque-forming units per mL (PFU·mL^−1^).

### 3.3. Flow Cytometry Assay

HK-2 cells were seeded in 6-well plates and infected with ZIKV at a multiplicity of infection (MOI) of 1. Cells were harvested at 24, 48, and 72 hpi, fixed with 3.7% PFA for 15 min, permeabilized with 0.15 % Triton X-100 in PBS for 5 min, and then blocked with PBS-BSA for 10 min. Cells were labeled with anti-E monoclonal antibody 4G2 (1/500) for 1 h at room temperature. Goat anti-mouse Alexa Fluor 488 IgG was used as the secondary antibody (1/1000) for 30 min. Cells were then subjected to a flow cytometric analysis using a CytoFLEX flow cytometer (Beckman Coulter, Brea, CA, USA). Results were analyzed using CytExpert software.

### 3.4. Immunofluorescence Assay

HK-2 were fixed (PFA, 3.7%) at 24, 48 and 72 hpi and permeabilized for 5 min (PBS 1× 0.15% Triton X-100). Cells were then stained for ZIKV using 4G2-Alexa Fluor 596 (1:1000 in PBS-BSA 2%) in the dark for 1h at room temperature. Mouse monoclonal antibody J2 was used to detect ZIKV double-stranded RNA (dsRNA). The goat anti-mouse Alexa Fluor 488 IgG was used as the secondary antibody (1/1000) for 30 min. The cell nuclei were delineated using DAPI staining. Coverslips were mounted in Vectashield and fluorescence was observed using a Nikon Eclipse E2000-U microscope. The images were captured using a Hamamatsu ORCA-ER camera and NIS-Element AR (Nikon) imaging software.

### 3.5. LDH Assay

Viral cytopathic effect was determined by Lactate Dehydrogenase (LDH) colorimetric assay measuring LDH release. Supernatants of ZIKV-infected cells were collected at 24, 48, and 72 hpi. LDH assay was performed using CytoTox 96 nonradioactive cytotoxicity assay (Promega, Madison, WI, USA) according to the manufacturer’s instructions. Absorbance was measured at 490 nm with background subtraction at 690 nm.

### 3.6. RT-qPCR

Quantification of ZIKV RNA was performed by RT-qPCR as previously described [44]. Briefly, total cellular RNA was extracted from cells with RNeasy kit (Qiagen, Hilden, Germany) according to manufacturer’s instructions and reverse transcribed using a specific E reverse primer (5′-TTCACCTTGTGTTGGGC-3′) and M-MLV reverse transcriptase (Life Technologies, Carlsbag, CA, USA) at 42 °C for 50 min. cDNA were amplified using 0.2 µM of each primer (forward primer: 5′-GTCTTGGAACATGGAGG-3′ and above described reverse primer) and 2X Absolute Blue qPCR SYBR Green Low ROX Master Mix (ThermoFisher, Waltham, MA, USA) on a CFX96 Real-Time PCR Detection System (Bio-Rad, Life Science, Hercules, CA, USA). The CFX96 program was used to calculate the threshold cycle (Ct) for each well in the exponential phase of amplification. A standard curve was obtained from a synthetic gene coding for nucleotides 954 to 1306 of ZIKV^MR766^ strain (GenBank: LC002520) cloned into pUC57 plasmid as previously described [22]. The standard curve was then used to calculate the absolute quantitation of viral RNA.

### 3.7. Virus Binding Assay

HK-2 cells grown in 12-well plates were pre-chilled at 4 °C for 30 min. Cells were incubated with ZIKV in DMEM containing 5.6 or 25 mM glucose at MOI of 1 at 4 °C for 1 h. The inoculum was then removed, and cells were washed with cold MEM supplemented with 2% FBS. Samples were then submitted to RT-qPCR. EGCG (100 µM) was used as a binding inhibitor.

### 3.8. Internalisation Assay 

HK-2 cells grown in 12-well plates were pre-chilled at 4 °C for 30 min. Cells were incubated with ZIKV^BR15^ and ZIKV^MR766^ in DMEM containing 5.6 or 25 mM glucose at MOI of 1 at 4 °C for 1 h and then shifted to 37 °C for another 1 h. Cells were washed with citrate buffer, to eliminate the bound noninternalized virus, and subjected to RT-qPCR. Q3G (200 µM) was used as an internalization inhibitor.

### 3.9. Fusion Assay

HK-2 cells were grown in 12-well plates. Pre-chilled cells at 4 °C for 30 min were inoculated with ZIKV in DMEM containing 5.6 or 25 mM glucose for 1 h at 4 °C. Then the temperature was shifted to 37 °C for 1 h. Next, 100 µM chloroquine were added for 2 h at 37 °C. Cells were then washed twice with PBS to eliminate the cytotoxic effects of chloroquine and fresh mediums were added. Cells were further incubated up to 30 h post temperature shifting. Total cellular RNA was extracted with an RNeasy kit and submitted to RT-qPCR.

### 3.10. MTT Assay

MTT (3-[4-dimethylthiazol-2-yl]-2,5-diphenyltetrahzolium bromide) colorimetric assay was used to evaluate cellular metabolic activity. HK2 cells grown in 96-well plates were inoculated with ZIKV. At 24, 48, and 72 h hpi, culture supernatants were discarded and 20 µL of 5 mg/mL MTT solution (Sigma-Aldrich, France) were added to the cells. After 2 h at 37 °C, the MTT solution was discarded and formazan crystals were solubilized in 50 µL of dimethyl sulfoxide (DMSO). Absorbance was measured at 570 nm with background subtraction at 690 nm.

### 3.11. Transfection Experiments

Cells grown in 12 well-plates were transfected with different concentrations of plasmid expressing the reporter gene GFP (0.2, 0.4, 0.6, 0.8 and 1µg/mL) (Genecust). Transfections were performed with Lipofectamine 3000 (Invitrogen, Carlsbag, CA, USA), according to the manufacturer’s instructions. 24 h post-transfection, cells were washed twice with PBS, harvested, and subjected to a flow cytometric analysis using a CytoFLEX flow cytometer (Beckman Coulter, Brea, CA, USA). Results were analyzed using CytExpert software.

### 3.12. Statistical Analysis

Statistically significant differences between conditions were analyzed with two-way ANOVA test and paired student *t*-test. All values were expressed as mean ± SEM of at least three independent experiments. When applicable, statistical analyses have been made on kinetics rather than on individual time points. All statistical analyses were done using the software Graph-Pad Prism version 8.0 (GraphPad Software, San Diego, CA, USA). Degrees of significance are indicated on the figures as follows: * *p* < 0.05; ** *p* < 0.01; *** *p* < 0.001, ns = not significant.

## Supplementary Materials

The following are available online at https://www.mdpi.com/1422-0067/22/5/2495/s1, Figure S1. Growth kinetics of HK-2 cells under normal and high-glucose conditions; Figure S2. Osmolarity does not lead to ZIKV restriction in HK-2 cells; Figure S3. Protein translation in HK-2 cells is not affected under high glucose condition.

## Abbreviations

BSA	Bovine Serum Albumin
DAG	Angstrom (unit of length equal to 10^−10^ m)
DKD	Diabetic Kidney Disease
DMEM	Dulbecco’s Modified Eagle’s Medium
DMSO	DiMethyl SulfOxide
DNA	DeoxyriboNucleic Acid
dsRNA	double stranded RNA
EGCG	EpiGalloCatechin Gallate
ER	Endoplasmic Reticulum
FBS	Fetal Bovine Serum
GFP	Green Fluorescent Protein
HK-2	Human -Kidney-2
hpi	hours post-infection
IgG	Immunoglobulin G
LDH	Lactate DeHydrogenase
mAb	monoclonal Antibody
MEM	Minimum Essential Medium
MFI	a Fluorescent Intensity
MOI	Multiplicity of Infection
MTT	3-[4,5-dimethylthiazol-2-yl]-2,5-diphenyltetrazolium bromide
Nrf2	Nuclear factor erythroid 2–related factor 2
PBS	Phosphate-Buffered Saline
PFA	ParaFormAldehyde
PFU	Plaque Forming Unit
PKC	Protein Kinase C
Q3G	Isoquercitrin
RNA	RiboNucleic Acid
RT-qPCR	quantitative Reverse Transcription-Polymerase Chain Reaction
WNV	West Nile Virus
ZIKV	Zika Virus
ZIKV^BR15^	Zika Virus Brazilian strain
ZIKV^GFP^	Zika Virus GFP
ZIKV^MR766^	Zika Virus Uganda strain
