# Supplementary material for: Zika Virus Growth in Human Kidney Cells Is Restricted by an Elevated Glucose Level
Source: Int J Mol Sci. 2021 Mar 2;22(5):2495. doi: 10.3390/ijms22052495 (PMC7958337; doi:10.3390/ijms22052495)
Supplement: Supplementary file 1 [file ijms-22-02495-s001.pdf]

International Journal of
Molecular Sciences

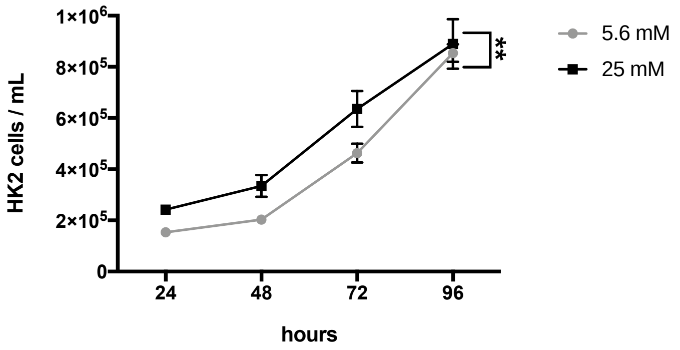

Supplementary Figure 1. Growth kinetics of HK-2 cells under normal and high-glucose conditions. HK-2 cells were seeded in 6-well plate at the same number of cells per well in normal and high-glucose culture medium. Cells were grown and counted over a 96 h period using standard hemocytometer. Results are shown as means ± SEM of three independent experiments. Two-way ANOVA test was used for statistical analysis (**p< 0.01).

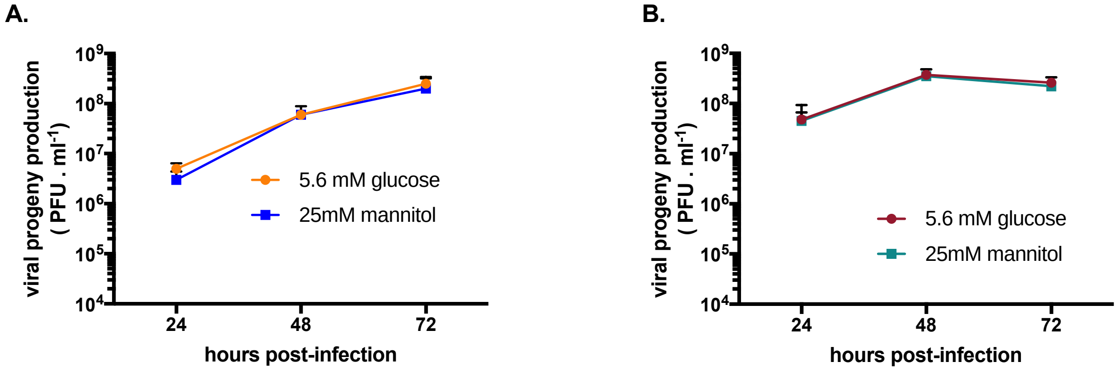

Supplementary Figure 2. Osmolarity does not lead to ZIKV restriction in HK-2 cells. HK-2 cells were high-mannitol adapted over a 10-day period to allow metabolic reprogramming, mimicking glucose reprogramming. Cells were first grown in 5.6 mM glucose + 10 mM mannitol medium for 3 days then shifted in 5.6 mM glucose + 20mM mannitol medium for another 7 days. HK-2 cells cultured under low-glucose (5.6 mM) and high-mannitol (5.6 mM glucose + 20 mM mannitol) conditions were infected at MOI of 1. ZIKV progeny production in cell culture supernatants of HK-2 infected cells with ZIKV^{BR15} (A) and ZIKV^{MR766} (B) were quantified by plaque-forming assay at 24, 48 and 72 hpi. Results are shown as means ± SEM of three independent experiments. Two-way ANOVA test shows that differences are not statistically significant (ns = not significant).

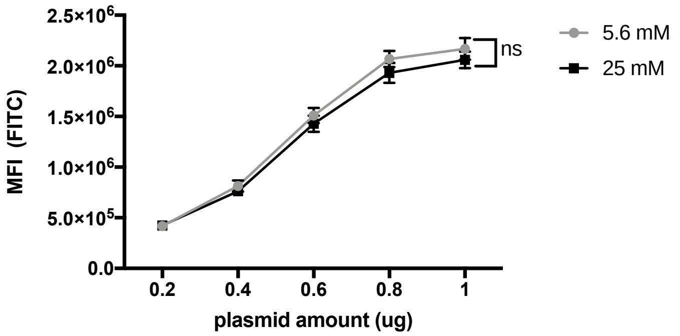

Supplementary Figure 3. Protein translation in HK-2 cells is not affected under high glucose condition. HK-2 cells were transfected with different amounts of pEGFP plasmid expressing the

reporter gene GFP (0.2, 0.4, 0.6, 0.8 and 1 amount/well). Transfection was performed using lipofectamine 3000. Cells were harvested 24 h post-transfection and submitted to flow cytometry analysis. Data were analyzed using CytExpert software and expressed as Mean Fluorescence Intensity (MFI). Results are shown as means ± SEM of three independent experiments. Two-way ANOVA test shows that the difference between these two conditions are not statistically significant (ns).
